# The Effect of Path and Beginning Time of Ascending on Incidence of Acute Mountain Sickness around Mount Damavand in Iran (5671 m)

**DOI:** 10.1155/2012/428296

**Published:** 2012-03-19

**Authors:** Reza Alizadeh, Vahid Ziaee, Lotf-Ali Frooghifard, Mohammad-Ali Mansournia, Ziba Aghsaeifard

**Affiliations:** ^1^Department of Anesthesiology, AJA University of Medical Sciences, Tehran, Iran; ^2^Sports Medicine Research Center, Tehran University of Medical Sciences, No. 7 Al-e-Ahamd Highway, P.O. Box 14395-578, Tehran, Iran

## Abstract

*Background*. This study was designed to evaluate the incidence of acute mountain sickness (AMS) occurring on different climbing routes on Mount Damavand and the effect of beginning time of ascent in Iranian trekkers. *Methods*. This study was a descriptive cohort investigation, performed in summer 2007. All trekkers who ascended Mount Damavand from northern, western, eastern, and southern paths and passed 4200 m altitude were included in the study. Two questionnaires were completed for each trekker (personal information and Lake Louise score questionnaire). Multiple logistic regression analysis was used to explore the independent predicting variables for AMS. *Results*. Overall incidence rate of AMS was 53.6%. This rate was the highest in south route (61.5%) (*P* < 0.001). There was no difference in the incidence of AMS on other paths. AMS history, AMS history on Damavand, the beginning time of climbing, sleeping at 4200 m altitude, and home altitude had significant effect on AMS incidence, but by multiple logistic regression analysis south route and AMS history on Mount Damavand had positive effect on incidence of AMS (*P* = 0.019 and *P* < 0.001). *Conclusion*. The path and the beginning time of ascent can affect incidence of AMS. The risk of occurrence of AMS was 1.9 times as large for trekkers who ascended from southern route.

## 1. Introduction

Acute mountain sickness (AMS) is a disorder which is seen in ascent to altitudes higher than 3000 meters. It is a clinical syndrome with headache and one or more other symptoms including gastrointestinal symptoms (poor appetite, nausea, vomiting), fatigue and/or weakness, dizziness/lightheadedness, and sleep disturbances [1, 2]. Its diagnosis is based on environmental symptom questionnaires, Hackett or Lake Louise AMS score [3, 4]. Several studies in different countries have been done so far, and incidence rates have been reported from 25% to 69% [2, 5–11]. Some effective factors have been considered in several studies, such as change of altitude related to residence altitude [3, 5, 11], speed of mountain climbing [1, 2, 11], very low and very high range of ages [5, 12], positive history of mountain sickness [8, 11], beginning mountain climbing at night [11], sleep in altitudes more than 3 hours in altitude [1], and water and fluid intake [13]. However, the relation of some factors like age and gender to the incidence of the disease is in doubt. 

Mount Damavand has four main paths for mountaineers' ascent. In our previous study, the incidence rate of disease was seen in more than 60% of mountaineers who ascend from the south [11]. Iranian expert mountaineers believe that ascent from southern path is accompanied by higher disease occurrence, but there is no documented data for this belief. On the other hand, some of the trekkers plan the beginning of mountain climbing at night. In the literature review, we did not find any investigation on beginning of ascending time (except our previous study [11]) and effect of path on incidence of AMS in other altitudes. So we designed this study for evaluating the incidence of AMS in different paths of Mount Damavand and the effect of beginning time of ascent on AMS incidence in Iranian trekkers.

## 2. Subjects and Methods

This study was a descriptive cohort study which was performed on trekkers of Mount Damavand during 8 weekends in summer 2007. Mount Damavand, Iran's highest mountain is located in the Alborz mountain range and it has special characteristic such as dormant volcano and single peak. All trekkers who climb over 4200 m were included in this study. Two questionnaires were completed for each trekker. These questionnaires were used in our previous study in Damavand. The first questionnaire was related to personal information and ascent characteristics (including sex, age, and home altitude, medical history, past history of AMS, beginning time of mountain climbing, number of acclimatization programs before this trekking, and duration of sleeping at over 4200 m), and the second questionnaire was Lake Louise score questionnaire. AMS was defined as a Lake Louise score more than 5 (Table 1) [3]. Sleeping more than 3 hours was considered as a risk factor for AMS. All of the questionnaires were completed by interview after taking informed consent. Interviewers were trained physicians when trekkers came back to the camps of 4200 m. 

According to our previous study [11], sample size was calculated to be 280 trekkers in all four paths (70 trekkers for each path). In this period, all men and women who ascended Mount Damavand from northern, western, eastern, and southern paths and passed 4200 m altitude were included in the study. Exclusion criteria included mountaineers who had used drugs for AMS prevention (acetazolamide, dexamethasone, or aspirin) or individuals who could not ascend to the top because of climbing-related injuries. Convenient sampling was done with the presence of research team at the altitude of about 4200 meters in different paths. According to our previous experience [11], the participants were divided into three groups by their age, under 15 y, 16–50 y, and over 50 y. Trekkers were divided into 3 groups based on the number of ascents they had made to altitudes above 4000 m in the last 6 months: experienced (more than 3 ascents), semiexperienced (1-2 ascents), and inexperienced (less than 1 ascents).

This study was approved by research committee of Sports Medicine Research Center and ethics committee of Tehran University of Medical Sciences. 

The collected data were analyzed by SPSS (version 15). Qualitative data were summarized as frequencies, whereas continuous data were expressed as mean and standard deviation. Categorical variables were analyzed with the chi-square and Fisher exact test and numerical variables were analyzed with the independent *t*-test. Bonferroni correction was used for multiple comparisons between the groups. Multiple logistic regression analysis was used to explore the independent predicting variables for AMS. For inferential purpose, 95% confidence interval was presented for the estimated effects (e.g., odds ratio). A 2-sided *P* value less than 0.05 was considered significant.

## 3. Results

From 360 completed questionnaires, 9 persons were excluded because of drug utilization and none of the studied persons was injured and finally 351 persons were studied (including 300 male and 51 female Mountaineers). Mean age of participants was 35.08 years, and its range was from 12 to 69 years. AMS occurred in 188 trekkers, so the overall incidence rate of AMS was 53.6%. The mean age of patients and nonpatients were 35.3 and 34.8 years, respectively. This difference was not statistically significant (*P* = 0.9). The incidence rate under 15 y, 16–50 y, and over 50 y was 33.8%, 48.5%, and 44.2%, respectively, but these differences were also not statistically significant (*P* = 0.7). Forty-nine percent of male participants and 41.2% of female participants were affected, and this difference was also not statistically significant (*P* = 0.2). 

The highest incidence rate of AMS was in southern path.  Table 2 shows the number of mountaineers and the incidence of AMS in each path. The difference between the incidences among different paths was highly significant (*P* < 0.001). Although AMS incidence was significantly higher in southern path in comparison to others, there was no difference between the incidences of AMS in other paths (Table 3). Seventeen to thirty-one percent of trekkers in north, west, and east paths were inexperienced, while this percent in south path was about 47%. There is no significant difference between experience of trekkers in north, west, and east paths, but this variable was significantly different in south path in comparison to others (*P* = 0.01). 

The minimum incidence of the disease as it is shown by Table 4 was in individuals who started climbing between 6 and 12 AM (*P* = 0.02), but the time of mountain top arrival had no influence on the disease incidence (*P* = 0.4). Occurrence of disease was greater in persons who did not have acclimatization. This difference tended to be statistically significant (*P* = 0.06). In any case the number of acclimatization programs, influencing the incidence rate, decreases. The mean acclimatization programs in nonpatients were greater or equal to 2.17 (SD = 1.46), while in patients were less or equal to 1.76 (SD = 1.25), and statistically significant (*P* = 0.025, CI = 0.05–0.76). Cigarette smoking and positive family history of AMS had no statistically significant influence on the incidence of the disease (*P* = 0.5 for each one).

Table 4 shows the incidence of disease predictors in the univariate analysis.  Table 5 shows multiple logistic regression models to identify determinants of AMS. Initial model includes beginning time of mountain climbing, sleeping at 4200 m altitude, home altitude, AMS history, and AMS history in Damavand. Multiple logistic regression analysis reveals that the southern path and AMS history on Mount Damavand increases the incidence of AMS in Damavand (*P* = 0.019 and *P* < 0.001, resp.). The risk of AMS occurrence for trekkers who ascended from southern path was 1.9 (CI = 1.1–3.3) (Table 5).

## 4. Discussion

The main goal of this study was assessment of acute mountain sickness incidence in different paths of Damavand. According to our review, this is the first study to evaluate effect of ascending path on AMS. The novel finding of this study is that the incidence of AMS had association with ascending route.

As a whole, the incidence rate of AMS in this study and in all four paths was the highest among studies on AMS. In previous studies, the incidence of AMS is reported as 68% in the study of mountaineers who climbed over 4200 m in Himalaya [5] and 69% in professional astronomy staff on Mauna Kea [9]. In other studies at about 4800 m or less which were mainly on Alps and Rocky mountains climbers, the incidence rate was lower than 40% [2, 5, 10, 14, 15]. As a whole, high incidence rate of AMS in our study can be due to specific characteristics of Mount Damavand or genetic characteristics of Iranian trekkers. The incidence of AMS was 10.4 to 34.5% in a study on Mount Tochal (another mountain in the Alborz range at 3450 m) [15, 16]. 

In our previous study which was also reported on the southern path of Damavand, incidence rate was 60.8% [11]. Our findings in this study showed that the incidence rate of AMS was unequal in difference routes of mountain. This finding confirms the expert climbers' idea about Damavand. They believe that southern route causes higher rate of AMS. Our study showed that AMS in southern path climbers was significantly higher than any other path, but there is not significant difference among any other paths. By multiple logistic regression analysis, we showed southern path has positive effect on AMS incidence rate.

A hypothesis is that the individual characteristics of Damavand in southern path (such as higher substantial amount of malodorous sulfur compounds and high-steep path in comparison with other paths) have caused this [11]. Some authors suggest that sulfur has a positive effect on incidence of AMS [17]. The expert climbers that have a prolong experience on ascending Damavand confirm it. Furthermore, a study on sulfur concentration in each path of Mount Damavand needs to be done. On the other hand, the southern route has less distance and it is the easiest route, so trekkers can ascent faster and less-experienced trekkers select this rout for ascending (as it is seen in Section 3). Faster mountain climbing and higher-inexperienced trekkers in south route can be other factors that influence higher rate of AMS in this route.

Often trekkers believe the best time for trekking on the Damavand is at the early morning (before 4 AM), and this time is accompanied by lower AMS rate. We tested this hypothesis in this study. According to our findings, the lowest incidence of AMS was in trekkers who start climbing between 6 AM and 12 MD. It was similar with our findings in previous study [11]. This finding needs more investigation in the future and in other mountain tops. If it was found in the Damavand, it may be due to environmental factors (such as the higher concentration of sulfur during night period), and if it was found in other mountains, it may be due to personal character (such as fatigue at the night). Exercise in a fatigue person is accompanied by exacerbation of arterial hypoxemia [18]. 

Meaningful influence of positive past history of AMS on frequent AMS has been reported in other studies [5, 10, 11]. Influence of physiologic and genetic characteristics of individual suffering from AMS are guessed as important factors [19, 20]. However, in this study there was an association between AMS and positive history of AMS in the univariate analysis, but not in multiple logistic regression analysis. On the other hand, AMS history in Damavand was a positive factor for AMS occurrence in both the univariate and multiple logistic regression analyses. This finding confirms the similar finding in our previous study [11], and the Iranian expert trekkers think that there is a specific character in Mount Damavand which is such as high sulfur gas concentration in the air. 

Similar to our previous study and other studies, there was no relation between sex, age, and smoking and incidence rate of AMS [1, 11, 21]. However, a higher incidence of AMS has been reported in children and a lower rate in the elderly [1, 5, 22, 23]. The finding of this study in relation to the role of residence altitude in the incidence rate of the disease was the same as other studies [5, 11]. Speed of mountain climbing was an effective factor in the occurrence of AMS in other studies [1, 2, 22, 24], but in our study there was no meaningful difference between mean climbing time in patients and healthy groups.

Some study showed the effect of acclimatization in prevention of AMS [25]. Similarly, in our study we found that acclimatization programs cause more decrease in AMS. 

In this study like the previous one, there is a significant inverted relationship between sleeping in altitudes and the occurrence of AMS [11]. Fatigue due to short time of sleeping may be the reason for higher AMS, while insomnia is a symptom of AMS. However, the average time of sleep in affected and nonaffected individuals had no statistically significant difference. It seems that other factors such as experience of climbers or fatigue affect this finding. In our study, we did not find any association between sleep in altitude and incidence of AMS in multiple logistic regression analysis. 

None of the factors such as sleeping at 4200 m altitude, home altitude, and AMS history was significant predictors of AMS (southern path as a significant predictor of AMS doubles its risk in comparison to other routes).

## 5. Strength and Limitation

Our study was performed on Mount Damavand with the characteristics such as dormant volcano, single peak, and sulfur odors especially on south route. On the other hand, we did not find other study in this mount except our previous study. Moreover, no previous studies have evaluated the influence of the time of beginning mountain climbing on occurrence of AMS. 

Our study has some limitations. The incidence of AMS above 4200 m, this rate might be higher if climbers/trekkers who started climbing but turned around below 4200 m were included. In addition, we did not consider the rate of ascending in our subjects. It was different in different routes due to distance or difficult pathway. These items could influence the incidence of AMS.

## 6. Conclusion

Although several known factors cause AMS, there are also some unknown factors which do the same. Besides other factors, the path and the beginning time of climbing can affect the incidence of AMS. The risk of AMS occurrence was doubled for trekkers who ascended from southern path. In our study AMS history in general, AMS history on Damavand in particular, the beginning time of climbing, sleeping at 4200 m altitude, and home altitude had significant effect on AMS incidence.

## Figures and Tables

**Table 1:** Lake Louise score for detecting acute mountain sickness.

Self-report questionnaire	1. Headache	0 No headache
		1 Mild headache
		2 Moderate headache
		3 Severe headache, incapacitating
	2. Gastrointestinal symptoms	0 No gastrointestinal symptoms
		1 Poor appetite or nausea
		2 Moderate nausea or vomiting
		3 Severe nausea and vomiting, incapacitating
	3. Fatigue and/or weakness	0 Not tired or weak
		1 Mild fatigue/weakness
		2 Moderate fatigue/weakness
		3 Severe fatigue/weakness, incapacitating
	4. Dizziness/lightheadedness	0 Not dizzy
		1 Mild dizziness
		2 Moderate dizziness
		3 Severe dizziness, incapacitating
	5. Difficulty sleeping	0 Slept as well as usual
		1 Did not sleep as well as usual
		2 Woke many times, poor nights sleep
		3 Could not sleep at all
Clinical assessment	6. Change in mental status	0 No change in mental status
		1 Lethargy/lassitude
		2 Disorientated/confused
		3 Stupor/semiconscious
	7. Ataxia (heel-toe-walking)	0 No ataxia
		1 Maneuvers to maintain balance
		2 Steps off line
		3 Falls down
		4 Can not stand
	8. Peripheral edema	0 No peripheral edema
		1 Peripheral edema in one location
		2 Peripheral edema in two or more locations

The Self-report score above (questions 1–5) stands alone, and this is recommended for general mountain travellers.

Additional observations are sometimes used by researchers.

The clinical assessment score (questions 6–8) can be* added * to the self-report score, in which case, in the context of a recent rise in altitude, a score of 5 or more would be taken as AMS.

AMS: altitude rise and headache and at least 1 other symptom (from Q1–5) and a total score of 5 or more (Q1–8).

**Table 2:** The incidence rate of AMS in trekkers in each path of Mount Damavand.

Path	Point of starting	No of climber	Incidence rate of AMS	*P* value
South	2800	143	61.5%	
North	2350	73	38.4%	<0.001
West	2900	64	45.3%	
East	2950	71	32.4%	

**Table 3:** Comparison between risk estimation of AMS incidence in different paths.

Different path	*P* value*	OR (odds ratio)	95% CI**
South to North	<0.001	2.57	1.44–4.59
South to East	<0.001	3.33	1.82–6.25
South to West	0.022	1.93	1.06–3.57
West to East	0.09	1.73	0.86–1.48
North to West	0.3	0.75	0.61–1.49
North to East	0.4	1.30	0.65–2.56

*P values are adjusted by Bonferroni correction.

**95% confidence interval for odds ratio.

**Table 4:** Variables associated with incidence of disease in the univariate analysis.

Variable	Definition	AMS		*P* value*
		Positive	Negative	
Beginning time of ascent^†^	00:00–05:59 06:00–11:59 12:00–17:59	106 (60.6%) 70 (44.9%) 10 (52.6%)	68 (39.4%) 86 (55.1%) 8 (47.4%)	0.02
Sleeping at 4200 m altitude	Positive Negative	139 (45.3%) 27 (67.5%)	168 (54.7%) 13 (32.5%)	0.01
Home altitude	Less than 1000 m More than 1000 m	33 (61.1%) 132 (44.9%)	21 (38.9%) 162 (55.1%)	0.03
AMS history	Positive Negative	79 (55.2%) 88 (42.7%)	64 (44.8%) 118 (57.3%)	0.02
AMS history in Damavand	Positive Negative	75 (56.4%) 61 (36.5%)	58 (43.6%) 106 (63.5%)	0.001

*Based on chi-square or Fisher exact test.

^†^There was just 1 participant who trekked between 18:00 and 11.59 PM. He did not meet criteria of AMS, so we omitted him from analysis of this item.

**Table 5:** Multiple logistic regression model to identify determinants of AMS.

Variable	*P* value	OR	95.0% CI for OR	
			Lower	Upper
Path	0.019	1.90	1.11	3.26
AMS history in Damavand	<0.001	2.90	1.70	4.94

Initial model includes beginning time of ascent, sleeping at 4200 m altitude, home altitude, AMS history, AMS history in Damavand, and number of ascents during previous 6 m.

## References

[1] Hackett PH, Roach RC (2001). High-altitude illness. *New England Journal of Medicine*.

[2] Roach RC, Loeppky JA, Icenogle MV (1996). Acute mountain sickness: increased severity during simulated altitude compared with normobaric hypoxia. *Journal of Applied Physiology*.

[3] Roach RC, Bartsch P, Hackett PH, Oelz O, Sutton JR, Coates G, Huston CS The lake louise AMS scoring consensus committee. The lake louise acute mountain sickness scoring system.

[4] Sampson JB, Cymerman A, Burse RL (1983). Procedures for the measurement of acute mountain sickness. *Aviation Space and Environmental Medicine*.

[5] Honigman B, Theis MK, Koziol-McLain J (1993). Acute mountain sickness in a general tourist population at moderate altitudes. *Annals of Internal Medicine*.

[6] Kayser B, Jean D, Herry JP, Bartsch P (1993). Pressurization and acute mountain sickness. *Aviation Space and Environmental Medicine*.

[7] Murdoch DR, Curry C (1998). Acute mountain sickness in the Southern Alps of New Zealand. *New Zealand Medical Journal*.

[8] Mistry G, Chandrashekhar Y, Sen U, Anand IS (1993). Study of acute mountain sickness during "rapid ascent" trekking to extreme altitude. *The Journal of the Association of Physicians of India*.

[9] Onopa J, Haley A, Mei EY (2007). Survey of acute mountain sickness on Mauna Kea. *High Altitude Medicine and Biology*.

[10] Wagner DR, Fargo JD, Parker D, Tatsugawa K, Young TA (2006). Variables contributing to acute mountain sickness on the summit of Mt Whitney. *Wilderness and Environmental Medicine*.

[11] Ziaee V, Yunesian M, Ahmadinejad Z (2003). Acute mountain sickness in Iranian Trekkers around mount damavand (5671 m) in Iran. *Wilderness and Environmental Medicine*.

[12] Basnyat B, Subedi D, Sleggs J (2000). Disoriented and ataxic pilgrims: an epidemiological study of acute mountain sickness and high-altitude cerebral edema at a sacred lake at 4300 m in the Nepal Himalayas. *Wilderness and Environmental Medicine*.

[13] Nerín MA, Palop J, Montaño JA, Morandeira JR, Vázquez M (2006). Acute mountain sickness: influence of fluid intake. *Wilderness and Environmental Medicine*.

[14] Maggiorini M, Müller A, Hofstetter D, Bärtsch P, Oelz O (1998). Assessment of acute mountain sickness by different score protocols in the Swiss alps. *Aviation Space and Environmental Medicine*.

[15] Jafarian S, Gorouhi F, Ghergherechi M, Lotfi J (2008). Respiratory rate within the first hour of ascent predicts subsequent acute mountain sickness severity. *Archives of Iranian Medicine*.

[16] Halabchi F, Mazaheri R (2008). Acute mountain sickness among overnight hotel guests: prevalence, symptoms and signs. *Tehran University Medical Journal*.

[17] Jaakkola JJK, Vilkka V, Marttila O, Jappinen P, Haahtela T (1990). The South Karelia air pollution study: the effects of malodorous sulfur compounds from pulp mills on respiratory and other symptoms. *American Review of Respiratory Disease*.

[18] Roach RC, Maes D, Sandoval D (2000). Exercise exacerbates acute mountain sickness at simulated high altitude. *Journal of Applied Physiology*.

[19] Zhou F, Wang F, Li F (2005). Association of hsp70-2 and hsp-hom gene polymorphisms with risk of acute high-altitude illness in a Chinese population. *Cell Stress and Chaperones*.

[20] Jiang CZ, Li FZ, He MA (2005). Glutathione S-transferase M1, T1 genotypes and the risk of mountain sickness. *Zhonghua lao Dong wei Sheng Zhi ye Bing za Zhi*.

[21] Schneider M, Bernasch D, Weymann J, Holle R, Bärtsch P (2002). Acute mountain sickness: influence of susceptibility, preexposure, and ascent rate. *Medicine and Science in Sports and Exercise*.

[22] Porcelli MJ, Gugelchuk GM (1995). A trek to the top: a review of acute mountain sickness. *Journal of the American Osteopathic Association*.

[23] Moraga FA, Osorio JD, Vargas ME (2002). Acute mountain sickness in tourists with children at Lake Chungará (4400 m) in northern Chile. *Wilderness and Environmental Medicine*.

[24] Broome JR, Stoneham MD, Beeley JM, Milledge JS, Hughes AS (1994). High altitude headache: treatment with ibuprofen. *Aviation Space and Environmental Medicine*.

[25] Lyons TP, Muza SR, Rock PB, Cymerman A (1995). The effect of altitude pre-acclimatization on acute mountain sickness during reexposure. *Aviation Space and Environmental Medicine*.

